# An intricate case of sporadic pseudohypoparathyroidism type 1B with a review of literature

**DOI:** 10.20945/2359-3997000000316

**Published:** 2020-12-15

**Authors:** José Diogo Ramalho e Silva, Gustavo Filipe Melo Alves da Rocha, Maria João Martins Oliveira

**Affiliations:** 1 Centro Hospitalar de Vila Nova de Gaia/Espinho (CHVNG/E) Departamento de Endocrinologia e Nutrição Vila Nova de Gaia Portugal Departamento de Endocrinologia e Nutrição, Centro Hospitalar de Vila Nova de Gaia/Espinho (CHVNG/E), Vila Nova de Gaia, Portugal

## Abstract

Pseudohypoparathyroidism comprehends an assorted group of genetically rare disorders that share end-organ resistance to parathyroid hormone. Genetic and epigenetic modifications on guanine nucleotide-binding protein alpha-stimulating gene locus are the most common underlying mechanisms associated with pseudohypoparathyroidism. Biochemical and molecular analysis stratify pseudohypoparathyroidism into types 1A, 1B, 1C, and 2. We describe an unusual case of sporadic pseudohypoparathyroidism type 1B. A 34-year-old Caucasian woman was admitted to the emergency department, with persistent asthenia, limb paresthesias, and tactile hyposensitivity. Her physical examination, previous personal and family histories were unsuspicious, except for mild, intermittent and self-limited complaints of paresthesia during her two pregnancies, but no detailed workup was done. No typical features of Albright hereditary osteodystrophy were observed. The initial laboratory investigation showed elevated parathyroid hormone level (311.2 pg/mL), hypocalcemia (albumin-corrected serum calcium 4.3 mg/dL), hypocalciuria, hyperphosphatemia, hypophosphaturia, and vitamin D deficiency. Combined calcium, vitamin D, and magnesium supplementation was commenced, with symptomatic and analytical improvement. Albeit resolution of vitamin D deficiency, the patient relapsed with mild and intermittent lower limb paresthesias. Pseudohypoparathyroidism was confirmed by molecular identification of the 3-kb STX16 deletion. The treatment was readjusted, and one year later, symptomatic remission was attained. Clinical and biochemical features, and their respective course, along with lack of distinctive features of Albright hereditary osteodystrophy pointed to pseudohypoparathyroidism type 1B. A careful follow-up is needed to avoid complications and recurrence. Once correction of hypocalcemia and hyperphosphatemia is achieved, with no reported complications and recurrence, a good prognosis is anticipated, comparable to the general population.

## INTRODUCTION

In 1942, Fuller Albright and cols. (1) described a condition characterized by increased serum levels of parathyroid hormone (PTH) related to its end-organ resistance, accompanied by hypocalcemia and hyperphosphatemia. The authors denominated that constellation of biochemical features as “pseudohypoparathyroidism” (PHP). Those patients were described as a global “thickset figure”, with rounded facies, short stature, short neck, brachydactyly, heterotopic subcutaneous ossifications, and cognitive impairment, a composite physical phenotype lately termed as Albright hereditary osteodystrophy (AHO).

Nowadays, PHP encompasses a genetically heterogeneous group of rare disorders that share end-organ resistance to PTH. The majority are detected in childhood (2). Although data about prevalence of PHP has been published [Japan – 0.34 in 100.000 (3); Denmark – 1.1 in 100.000 (2)], real prevalence is unknown (4). Up to 90% of PHP or related disorders a molecular diagnosis is made (5). The most frequent culprit mechanisms are sporadic or autosomal dominantly inherited genetic mutations and/or epigenetic alterations involving guanine nucleotide-binding protein alpha-stimulating (*GNAS*) gene locus on chromosome 20q13.3 (6). *GNAS* complex locus encodes the α-subunit of the stimulatory G protein (Gsα), a key regulator of adenylyl cyclase signal transduction. Hence, PTH response by measurement of serum and urinary cyclic adenosine monophosphate (cAMP) levels permit to categorise patients into PHP type 1 (diminished cAMP response), and PHP type 2 (preserved cAMP response), notwithstanding both have impaired phosphaturic responses (4,7). The latter suggests that the defect is downstream to cAMP production in the PTH-mediated signal transduction pathway, but no specific genetic disruption has been found (8).

Molecular analysis classifies PHP type 1 (PHP1) into three distinctive entities, namely PHP type 1A (PHP1A), PHP type 1B (PHP1B), and PHP type 1C (PHP1C), although some overlaps may occur. PHP1A presents several heterozygous inactivating mutations affecting Gsα protein. PHP1C appears to represent a subgroup of PHP1A as they have clinical and biochemical similarities. However, PHP1C are solely related to *GNAS* mutations that affect receptor coupling, but not its basal activity (4,6,8).

The human *GNAS* locus suffers genomic imprinting by deoxyribonucleic acid (DNA) methylation. It contains four differentially methylated regions (DMRs) comprising the promoters of four alternative transcripts: exon A/B (*GNAS-A/B*: TSS DMR = A/B), neuroendocrine secretory protein 55 (*GNAS-NESP*: TSS DMR = NESP), extra-large stimulatory G protein (*GNAS-XL*: Ex1 DMR = *XL*), and GNAS antisense (*GNAS*-AS1/: TSS DMR = AS1) (7,9). α is biallelically expressed in most tissues, but merely in the proximal renal tubules of humans, and, to a lesser extent, in the thyroid, pituitary, and gonads, it is paternally imprinted, the so-called tissue-specific genetic imprinting. Other transcripts of the *GNAS* complex locus (exon *A*/*B, XL*α, and *A*/*S*) are paternally expressed. On the other hand, syntaxin-16 (*STX16*) and *NESP55* suffer biallelic and maternal expression, respectively (10).

As far as is known, all PHP1B patients exhibit loss of maternal-specific methylation at exon A/B, leading to biallelic expression of the transcript (11,12). This defect might play a role in the repression of Gsα protein in the proximal tubule, and ensuing isolated PTH renal resistance. However, it has also been reported partial thyroid-stimulating hormone (TSH) resistance (13). Conversely, PHP1A and PHP1C patients display multiple hormonal resistances (PTH, TSH, growth hormone-releasing hormone, and gonadotrophins). Thus, PHP1B is clinically and biochemically different from the other subtypes of PHP1 as it causes more restricted deficiency of Gαs protein. PHP1B lack distinctive features of AHO but patients may experience mild brachydactyly (7,14). The majority of PHP1B cases are sporadic. Up to 20% of cases are inherited in an autosomal dominant fashion, mainly due to 3-kb deletions within maternal allele of *STX16* gene and other *GNAS* upstream regions (5). The pathways how the aforementioned deletions lead to methylation defects in exon *A*/*B* are not well understood (7,15). Beyond exon *A*/*B*, in a majority of sporadic cases of PHP1B, other DMRs are additionally impaired. No molecular explanations to that have yet emerged (12). Up to 10% cases are caused by partial or complete paternal uniparental isodisomy involving the long arm of chromosome 20, which lead to a partial/complete deficiency of Gsα protein in maternally expressed tissues (5,16). A great proportion of sporadic PHP1B remains unresolved, and does not appear to be related to the *GNAS* locus. Emerging opinions have been in favour of the impact of environmental and exogenous factors on DNA methylation alterations observed on this subset of patients (17,18).

Herein, we report a rare case of sporadic PHP1B due to a *de novo* 3-Kb *STX16* deletion.

## CASE REPORT

A 34-year-old Caucasian woman was admitted to the emergency department (ED), due to a two-week duration of persistent asthenia, four-limb paresthesias, and tactile hyposensitivity. The physical examination was normal. The patient was normoponderal (body mass index 22.9 kg/m^2^). Chvostek and Trousseau signs were negative. She underwent broad biochemical analysis (Table 1). No other family member of the patient was diagnosed with thyroid or parathyroid disorders or disruptions of calcium homeostasis. She had no usual medication. The patient experienced a normal growth and development, with no clinical features of AHO. Her school performance was outstanding. She holds a master's degree in Medicine. She had no reported disturbances during embryonic and fetal development or anomalies at birth. The patient's daughters experienced a healthy growth without specific physical abnormalities. However, during both pregnancies, the patient had intermittent and self-limited complaints of paresthesias, but no specific workup was performed.

**Table 1:** Laboratory findings at the emergency department

Parameters	Patient's value	Reference range
Albumin-corrected serum calcium (mg/dL)	4.3	8.8-10.2
Phosphate (mg/dL)	5.5	2.7-4.5
Magnesium (mEq/L)	1.5	1.3-2.1
Parathyroid hormone (pg/mL)	311.2	15.0-65.0
25-hydroxyvitamin D (mmol/L)	36	62.5-200
Creatinine (mg/dL)	0.52	0.51-0.95
pH	7.42	7.35-7.45
Thyroid-stimulating hormone (μIU/mL)	0.95	0.27-4.2
Free T4 (ng/mL)	1.35	0.93-1.70
Free T3 (pg/mL)	3.25	2.57-4.43

Differential diagnosis between PHP and vitamin D deficiency was assumed, conjugating clinical and analytical investigations.

She was hospitalized, and promptly initiated calcium gluconate perfusion. Subsequently, etiological workup was carried out. Symptomatic resolution and analytical improvement were achieved. Biochemical analysis at discharge are described on Table 2. The patient was discharged with calcium carbonate 9 g/day, calcitriol 0.75 mcg/day, cholecalciferol 2,400 IU/day and 1.2 g elemental magnesium/day. She was referred to the endocrinology consultation, where additional assessment was performed (Table 3).

**Table 2:** Laboratorial evaluation at the hospital discharge

Parameters	Patient's value	Reference range
Albumin-corrected serum calcium (mg/dL)	7.2	8.8-10.2
Phosphate (mg/dL)	4.8	2.7-4.5
Parathyroid hormone (pg/mL)	156	15.0-65.0
Creatinine (mg/dL)	0.54	0.51-0.95

**Table 3:** Laboratorial evaluation at the endocrinology consultation

Parameters	Patient´s value	Reference range
Albumin-corrected serum calcium (mg/dL)	7.7	8.8-10.2
Phosphate (mg/dL)	4.7	2.7-4.5
Parathyroid hormone (pg/mL)	156	15.0-65.0
25-hydroxyvitamin D (mmol/L)	62	62.5-200
Creatinine (mg/dL)	0.54	0.51-0.95
24-h urinary calcium (mg/24h)	2	100-320
24-h urinary phosphate (mg/24h)	320	400-1300
Thyroid peroxidase antibody (IU/mL)	21	<34
Thyroid-stimulating hormone receptor antibody	0.8	≤1
Thyroglobulin antibody	73	<115

Despite correction of vitamin D levels, mild and intermittent lower limb paresthesias had returned. Parathyroid glands were not visualized on neck ultrasound. Dual-energy X-ray absorptiometry (DEXA) of forearm, lumbar vertebrae, and femur neck were normal. Genetic analysis showed the 3-kb *STX16* deletion, confirming PHP1B.

During follow-up, the treatment was readjusted, and symptomatic remission was accomplished. The patient is currently medicated with calcium carbonate 4.5 g/day, cholecalciferol 22,400 IU/month, calcitriol 1 mcg/day, and 0.52 g of elemental magnesium/day. Table 4 exhibits current biochemistry panel.

**Table 4:** Six-month follow-up laboratory results

Parameters	Patient's value	Reference range
Albumin-corrected serum calcium (mg/dL)	6.9	8.8-10.2
Phosphate (mg/dL)	5.0	2.7-4.5
Magnesium (mEq/L)	1.72	1.3-2.1
Parathyroid hormone (pg/mL)	277	15.0-65.0
25-hydroxyvitamin D (mmol/L)	64	62.5-200
Creatinine (mg/dL)	0.61	0.51-0.95
Thyroid-stimulating hormone (μIU/mL)	2.08	0.27-4.2
Free T4 (ng/mL)	1.08	0.93-1.70
Free T3 (pg/mL)	3.05	2.57-4.43

## DISCUSSION

In the present case, clinical and laboratorial features led us to raise suspicion of vitamin D deficiency or PHP. Hyperparathyroidism was in first instance ruled out as the patient presented hypocalcemia, low 24-hour urinary calcium, and normal renal function. After normalization of vitamin D levels, clinical and biochemical patterns persisted and, thus PHP became the main culprit. Then, straightforward investigation was readily carried out through genetic testing. Sequence analysis of *GNAS* exons 1 through 13 was the first step. No alterations were identified. Therefore, methylation analysis was subsequently performed. A single loss of methylation at *GNAS*-*A*/*B*: TSS DMR=*A*/*B* was revealed, suggesting PHP1B. Despite loss of methylation identifies an imprinting defect, its main cause remains indefinite. Hence, quantitative polymerase chain reaction (PCR) was executed to assess for deletions or duplications. A unique 3-kb microdeletion within maternal *STX16* allele was detected. Sporadic PHP1B was diagnosed in line with absence of family history, and an unremarkable objective examination, due to isolated renal PTH resistance.

Taking into account that a maternally inherited 3-kb deletion within STX16 is the main underlying cause of autosomal dominant PHP1B, the same concept does not apply to sporadic cases (14). In fact, as far as is known, there are only three cases published in the literature of *de novo* 3-kb *STX16* deletions. Mantovani and cols. (19) reported a *de novo STX16* deletion after examining genomic DNA from 10 patients with clinically confirmed sporadic PHP1B. Later in 2012, Turan and cols. (20) found a solitary loss of methylation at GNAS exon *A*/*B* and the 3-kb *STX16* deletion on two index cases with clinical and biochemical features of PHP1B. In one family, the patient's mother and his sister did not show any clinical, biochemical or molecular alterations. Both siblings presented the same maternal allele, but the index case had an allelic loss for microsatellite marker 261P9-CA1 within *STX16*, comprising a *de novo* mutation. In the other family, the index case, his mother, three of his five siblings, and a maternal uncle exhibited the 3-kb *STX16* deletion. The deceased maternal grandfather, his siblings, and other offsprings were also tested for the deletion. It was only detected in the grandfather, meaning that he was a carrier of a *de novo STX16* deletion.

While rare, there are reported cases of PHP1B patients with mild to moderate AHO phenotype, the more common being isolated brachydactyly (21,22). Other end-organ hormone resistance, such as TSH [in some series > 50% of PHP1B patients (11)], may take place with mildly elevated TSH levels, which was not the case in our patient.

In 2018, the first international Consensus Statement for the diagnosis and management of PHP and its spectrum of disorders (8) was published. Its main goal was to provide patients and practitioners a comprehensive guide to report detailed new evidence concerning their molecular characterization and precise management. Establishing a correlation with the current case, the diagnosis of PHP is primarily clinical and biochemical, including PTH resistance as a mandatory criteria independent of positive family history, among other major criteria. Until recently, resistance to PTH was definitively established through measurements of cAMP and urine phosphate following PTH infusion (the Ellsworth-Howard test) (23,24). However, this consensus (8) did not recommend performing the test on a daily basis practice, but might be relevant for research purposes. Thus, for practical concerns, it was not performed.

The main goals of treatment comprise attempting to re-establish and maintain normocalcemia and normophosphatemia while avoiding hypercalciuria. Therefore, the mainstay of therapy is active vitamin D compounds (calcitriol) and calcium supplementation (25). PTH levels should diminished as far as serum and urinary calcium levels allow (8).

The 2018 consensus statement (8) also recommends that serum biochemistry panel and urinary calcium excretion must be obtained every 6 months in asymptomatic patients or more regularly in symptomatic patients, and when vitamin D requires increased dosage, such as during acute illnesses, pregnancy and/or breastfeeding. Patients and their family members should be advised about clinical manifestations of hypercalcemia and hypocalcemia. Moreover, the patient should be routinely screened, treated, and monitored for other related endocrinopathies, chiefly hypothyroidism. Thyroid function tests accompanied by autoantibodies should be performed at diagnosis. Recognition of TSH resistance and its prompt management are advocated in all PHP patients. Afterwards, TSH levels should be checked yearly in adults.

Since PHP1B patients did not exhibit skeletal PTH resistance, a basal DEXA should be considered (4,26). Hyperparathyroid bone disease is a real possibility if PHP is left untreated (15).

During follow-up, if neurological signs become apparent, a brain CT scan is mainly required to rule out the presence of intracranial calcifications due to persistent hyperphosphatemia and the ensuing elevation of serum calcium-phosphorus product (8). A standard ophthalmic exam should be addressed to search for cataracts. Dental examinations should also be carried out.

Once PHP1B might be asymptomatic for generations, all PHP1B patients should be tested for the 3-kb *STX16* deletion even though with a negative family history (20). Regarding genetic counselling, guidance should be tailored to the diverse molecular findings. For a sporadic methylation defect involving the maternal allele, the index patient is uniquely affected, and further testing of other family members and future descendants is merely a matter of research interest (8).

Until now, there are no reported cases of assisted or complicated pregnancies involving sporadic PHP1B women (17). No data has arisen regarding lactation and timing of menopause (8).

In conclusion, current evidence suggests that sporadic cases of PHP1B due to *de novo* 3-kb *STX16* deletions are fairly unique (20). This case also showed that this condition may remain clinically silent for several years. Treatment of hypocalcemia and hyperphosphatemia and a careful follow-up are needed to avoid complications and recurrence of both. Once it is accomplished, a good prognosis is expected, and similar with the general population (2).
